# The relationship between group attachment and career adaptability of college students: a moderated mediation model

**DOI:** 10.3389/fpsyg.2025.1473147

**Published:** 2025-07-02

**Authors:** Huiyan Wang, Feng Lu, Shaoying Liu

**Affiliations:** ^1^College of Education, Zhejiang University, Hangzhou, China; ^2^Party Committee and Student Work Department, Zhejiang Sci-Tech University, Hangzhou, China; ^3^Department of Enrollment and Employment, Hangzhou Dianzi University, Hangzhou, China; ^4^Department of Psychology, Zhejiang Sci-Tech University, Hangzhou, China

**Keywords:** group attachment, career adaptability, perceived social support, academic achievement level, college students

## Abstract

**Introduction:**

This study explored the connection between group attachment and career adaptability among college students, considering attachment theory and career construction theory. It also looked into the mediating role of perceived social support and the moderating role of academic achievement level.

**Methods:**

The Career Adapt-Abilities Scale - China Form, The Group Attachment Scale, The Perceived Social Support Scale, and The Academic Achievement Level Questionnaire were used to survey 533 Chinese college students.

**Results:**

The survey results indicated that perceived social support partially mediated the relationship between group attachment and college students’ career adaptability. The relationship between perceived social support and career adaptability was significantly positively moderated by academic achievement level.

**Discussion:**

The study results improve the understanding of the factors affecting career adaptability and provide evidence for higher education institutions to enhance class group dynamics and boost college students’ employability.

## 1 Introduction

One of the crucial aspects of higher education is promoting graduates’ acquisition of working abilities and employment opportunities. In recent years, the phenomenon of “delayed employment” among college graduates has continued to rise, with the proportion of “delayed employment” among 2023 graduates reaching 18.9% (Wang, 2023). Career adaptability is defined as “the ability to adjust to predictable career tasks, occupational roles, or unpredictable changes in work and working conditions” (Savickas, 1997). Studies have found that career adaptability promotes career exploration (Neureiter and Traut-Mattausch, 2017) and enhances employability (Kwon, 2019). College students with low career adaptability face more difficulties in making career decisions (Parola and Marcionetti, 2021), and are more likely to experience “delayed employment,” “non-employment,” or even “unemployment.” Discussing the factors that influence college students’ career adaptability is of great significance in promoting high-quality employment for graduates. Investigating the factors that impact college students’ career adaptability is crucial for enhancing their overall development, fostering positive psychological traits and lifelong learning skills, and attaining the desired goals of talent cultivation in higher education.

Career adaptability is a key concept in career construct theory, referring to an individual’s psychological and social resources for dealing with current and future career tasks, transitions, and challenges (Savickas, 1997). According to career construction theory, these resources help individuals navigate the complexities of career development (Savickas, 2013). Adaptive readiness, as a precursor to career adaptability, has been studied in terms of personality traits, cognitive factors, and emotional factors (Rudolph et al., 2017a; Rudolph et al., 2017b; Lei et al., 2021). Furthermore, scholars have examined how family socioeconomic status (Zhao, 2012) and parents’ career-related behaviors (Zhao et al., 2022) can affect the career adaptability of college students by considering family environmental factors. However, there remains a gap in research regarding the impact of campus environmental factors on this aspect. Studying and living in class groups is crucial for college students, and it is essential to investigate how these groups impact individual career adaptability. Psychological perspectives have been recommended as a means to assist students in enhancing their learning performance and effectiveness at a micro level, thereby equipping them with the necessary literacies and competencies for the 21st century, according to Ye J. et al. (2023), Ye et al. (2023a), and Ye et al. (2023b). Group attachment is a reflection of how an individual feels about the group they belong to. It represents their self-group identity and is formed through interactions with the group and its members. This study aims to explore the impact of classroom group attachment on career adaptability within the campus environment, and to investigate how social support mediates the relationship between group attachment and academic achievement. This study seeks to explore how the emotional attachment level among college students and their class groups, the development of a strong social support system, and a varied academic achievement evaluation system can improve college students’ career adaptability.

### 1.1 Group attachment and career adaptability

Improving college students’ ability to adapt to the future workplace and helping them transition smoothly from school to their careers is a key aspect of quality higher education and a shared objective for students as part of their academic community. Group attachment is the emotional connection and identification that an individual has with a group (class), indicating their general liking or disliking of that particular group (class). This emotional bond is enduring and consistent over time (Li and Liu, 2012). Although there is a significant correlation between group attachment and adult attachment, there are differences between the two. College students tend to have both secure adult attachments and insecure group attachments (Smith et al., 1999). Secure group attachment has three main functions: it offers individuals a readily available source of attachment figures (other group members) during times of need, provides support and comfort during stressful situations, and helps in the development of social, emotional, and cognitive skills through group activities (Rom and Mikulincer, 2003). Group attachment is often defined by measuring the dimensions of group attachment anxiety and avoidance. Group attachment anxiety is when someone worries about being accepted by the group, while group attachment avoidance is when someone tends to avoid depending on the group. Low scores on both dimensions indicate a secure group attachment, while high scores on both dimensions indicate an insecure group attachment. Research has shown a significant negative relationship between group attachment and school adjustment among college students, even after taking their level of adult attachment into account (Marmarosh and Markin, 2007). Group attachment theory suggests that a secure attachment to a group can help individuals develop a positive intra-group working model. This model provides individuals with the internal resources needed to actively participate in group activities. Conversely, an insecure group attachment can lead to a negative intra-group working model, discouraging individuals from engaging in activities like career development class meetings, alumni lectures, and employment salons, all of which are crucial for career exploration. Studies have shown that attachment, as a personal trait, can greatly influence how individuals approach career exploration and decision making (Blustein et al., 1991). Specifically, insecure attachment has been found to be a strong predictor of low career adaptability (Ramos and Lopez, 2018). Therefore, we propose the following:

*Hypothesis 1*: Group attachment will be negatively associated with career adaptability.

### 1.2 Mediating role of perceived social support

In order to delve deeper into how group attachment impacts career adaptability, it is essential to explore the mediating factors that connect the two. Perceived social support is when an individual believes and evaluates that they are supported, understood, and respected by the outside world (Sarason et al., 1991). College students rely on support systems such as family, friends, and groups to help them navigate the challenges of college (Ye J. et al., 2023; Ye et al., 2023a; Ye et al., 2023b). These groups provide essential instrumental, informational, and emotional support to assist with academic and social adaptation (McLean et al., 2022; Ye J. et al., 2024; Ye J. H. et al., 2024). According to a study, the perceived social support of college students plays a crucial role in predicting their career adaptability (Su et al., 2016; Salim et al., 2024; Zhang, 2019). Additionally, the study revealed that group attachment was a strong predictor of reduced social support (Smith et al., 1999). College students who have an insecure group attachment tend to have negative perceptions and expectations regarding support from the group and its members, which can hinder their psychological and social resources for exploring career options. Therefore, we propose the following:

*Hypothesis 2*: Perceived social support will play a mediating role between group attachment and career adaptability.

### 1.3 Moderating role of academic achievement level

Academic achievement level, which are typically determined by an individual’s class rank and grades, play a crucial role in shaping their career development (Hou, 2019). High academic achievers demonstrate greater adaptability in college settings (Li et al., 2022), while individuals with low academic performance tend to struggle more with psychological challenges in college (Townsend et al., 2019). Students who achieve high academic levels tend to have stronger motivation and set higher goals for their academic and career development. This future-oriented mindset helps individuals actively explore themselves and their surroundings, leading to a significant correlation with career adaptability (Hsieh, 2025) and a positive prediction of career adaptability (Datu and Buenconsejo, 2021). Simultaneously, college students exhibit varying cognitive (Strobel et al., 2019) and affective experiences (Camacho-Morles et al., 2021) based on their academic achievements, leading to distinct sensitivities to perceived social support. Research also indicates that college students with varying academic achievements exhibit differences in behaviors such as parental academic involvement and stress perception (Zhang et al., 2021). College students who achieve high academic success tend to perceive higher levels of social support (Ye et al., 2014). Based on the protective-protective model of human development, various protective factors can combine to influence developmental outcomes (Fergus and Zimmerman, 2005; Wang et al., 2009). For instance, the level of academic achievement might impact the connection between perceived social support and career adaptability. Therefore, we propose the following:

*Hypothesis 3*: Academic achievement level will play a moderating role between perceived social support and career adaptability.

**Figure 1:**
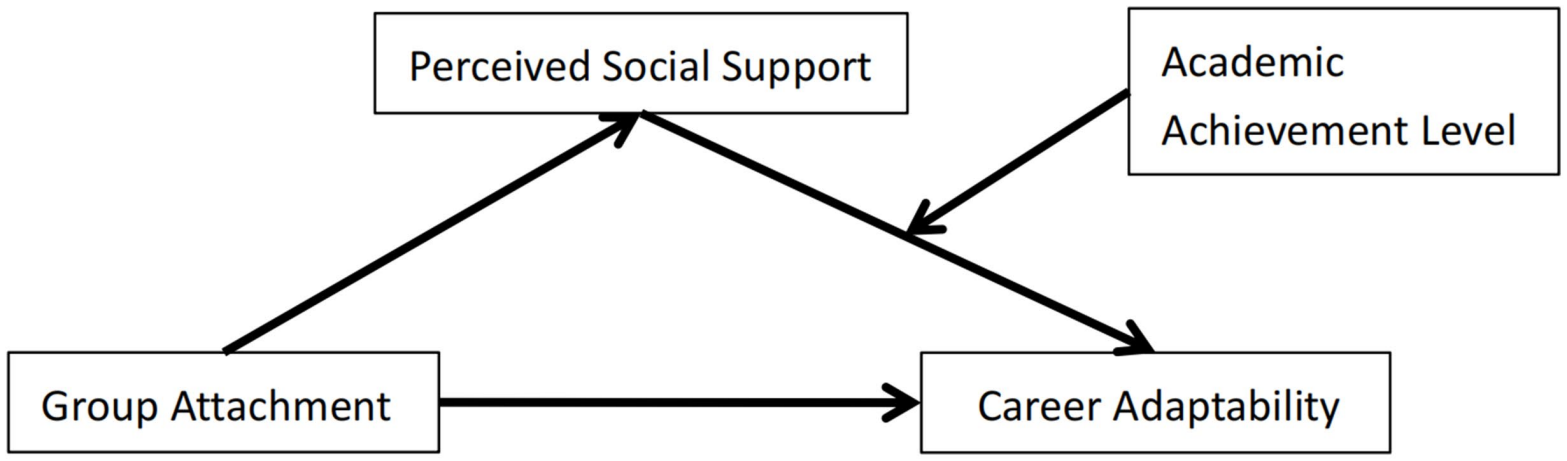
Hypothetical research model.

According to the construct model of “adaptive readiness-career adaptability” of career construct theory, group attachment can significantly and negatively predict career adaptability. It can also significantly predict career adaptability through the mediating role of perceived social support, while academic achievement level plays a moderating role between perceived social support and career adaptability. A hypothesized model was developed, as shown in Figure 1.

## 2 Methods

### 2.1 Participants

A cluster random sampling method was utilized to choose 533 students from a college in Zhejiang, China. The survey was conducted using the online platform Wenjuanxing, and a total of 533 questionnaires were collected. After removing 91 invalid questionnaires, there were 442 valid questionnaires (82.9%). Among the respondents, there were 189 male students (42.76%) and 253 female students (57.24%). In terms of academic years, there were 86 freshmen (19.46%), 137 sophomores (31%), 100 juniors (22.62%), and 119 seniors (26.92%). Additionally, 236 (53.39%) students majored in science and engineering, 62 (14.03%) students majored in literature and history, and 144 (32.58%) students majored in art. The age range was between 17 and 23 years old, with an average of 19.92 and a standard deviation of 1.28.

This study received approval from the Psychology Ethics Committee of Zhejiang Sci-Tech University (No. 202109×002), and all participants provided informed consent.

### 2.2 Measures

*Career adaptability* was assessed using the Career Adapt-Abilities Scale—China Form (Hou et al., 2012). There are a total of 24 questions divided into four subscales: Career Concern, which includes questions like “Preparing for the future”; Career Control, which includes questions like “Making decisions by myself”; Career Curiosity, which includes questions like “Becoming curious about new opportunities”; and Career Confidence, which includes questions like “Solving problems.” Participants rated each item on a 5-point scale (1 = not strong, 5 = very strong), with higher scores indicating greater career adaptability. The overall Cronbach’s alpha coefficient of the scale was 0.97.

*Group Attachment* was evaluated using The Group Attachment Scale (Li and Liu, 2012), which includes two subcategories: group attachment anxiety and group attachment avoidance, with 18 questions in each category. Examples of questions are “I worry that I will be abandoned by the group” and “I tend not to get too close to the group.” Participants rated each question on a scale from “1 = completely disapprove” to “7 = completely disapprove,” with higher scores indicating higher levels of insecure attachment to the group. Individuals with higher scores are more likely to have higher levels of insecure attachment to the group. The Cronbach’s alpha coefficient for the scale in this study was 0.89.

*Perceived Social Support* was measured using the Perceived Social Support Scale (Jiang, 1999), which includes 12 self-reported items rated on a scale from “1 = strongly disagree” to “7 = strongly agree.” Examples of questions are, “My friends can really help me.” A higher score indicates a greater overall sense of social support experienced by the individual. The Cronbach’s alpha coefficient for the scale in this research was calculated to be 0.94.

*The Academic Achievement Level Questionnaire* was used to measure academic achievement level, which is determined by comparing an individual’s academic results with others in the learning process (Zaugg, 1998). This evaluation helps assess the learner’s own learning level and ability (Xu, 2005). Scholars categorize the academic achievement of subject samples into three dimensions: academic performance, ability development, and self-concept development. Academic performance pertains to students’ course examination results, ability refers to qualities beyond examination results, and self-concept relates to the experience and perception of self-existence. Moreover, the data on academic achievement is gathered through self-assessment by the samples (Jia et al., 2020). Hence, this research utilized a self-administered questionnaire to assess academic achievement levels, with options including: 1 = “Bottom 30%,” 2 = “Middle,” 3 = “Top 30%.” A higher score indicates a higher level of academic achievement. Chinese colleges and universities usually conduct a comprehensive assessment of students’ professional performance, classroom performance, physical exercise, social practice, and innovative ability. The results of this assessment are shared within a certain range, providing a basis for sophomores, juniors, and seniors to accurately evaluate their academic achievement levels. First-year students can assess their academic achievement in segments by combining their college entrance exam scores with their school performance. First-year students can assess their academic achievement level by segments using their college entrance examination scores and school performance. The researcher observed that the segmented self-assessment results matched the actual situation.

### 2.3 Data analysis

Descriptive statistics and correlations were first analyzed using SPSS version 23.0, and a one-way validated factor analysis was conducted with Mplus 7.4 socio-statistical software. Second, we used the PROCESS macro (Model 4) of SPSS software to examine the indirect effect of perceived social support in linking group attachment and career adaptability. The bias-corrected bootstrapping method, based on 5,000 samples, was used to test the significance of the indirect effect. Third, we conducted a moderated mediation analysis using PROCESS (Model 14) to determine whether the indirect path was moderated by academic achievement level. Finally, we calculated conditional indirect effects to further test whether the indirect effect varied under different values of the moderating variable.

## 3 Results

### 3.1 Common method bias test

The Harman one-way test was utilized to perform exploratory factor analysis, identifying 12 factors with eigenvalues greater than 1. The variance explained by the initial factor was 27.31%, which did not meet the critical value of 40%. The results of the one-way validation factor analysis conducted using Mplus 7.4 software indicated a poor model fit with χ^2^/df = 6.43, CFI = 0.43, TLI = 0.41, RMSEA = 0.11, and SRMR = 0.16. This suggests that all items in the assessment should not be grouped under the same factor, leading to the conclusion that serious common method bias was not present in this study.

### 3.2 Descriptive statistical analysis

The descriptive statistics and correlation analysis results can be seen in Table 1. Despite controlling for gender and grade variables, group attachment was found to have a significant negative correlation with perceived social support and career adaptability. Additionally, there was a borderline positive correlation between group attachment and academic achievement level (*p* = 0.06). There was a significant positive correlation between perceived social support and career adaptability, as well as between career adaptability and academic achievement level. Perceived social support also showed a significant positive correlation with academic achievement level at a *p*-value of 0.03.

**Table 1:** Mean values, standard deviations and correlation coefficients of variables (*N* = 442).

Variables	*M*	SD	1	2	3	4
1. Academic achievement level	2.28	0.66	1			
2. Group attachment	3.65	0.59	0.09	1		
3. Perceived social support	5.28	0.95	0.11*	−0.33***	1	
4. Career adaptability	3.8	0.63	0.19***	−0.28***	0.43***	1

*p < 0.05, **p < 0.01, ***p < 0.001.

### 3.3 Examining the mediation model

**Figure 2:**
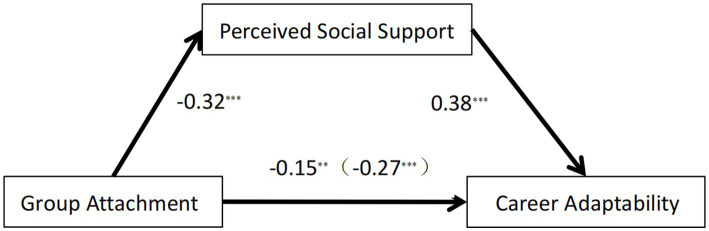
The mediating effect of perceived social support in the association between group attachment and career adaptability. **p < 0.01, ***p < 0.001.

The mediating effect of perceived social support between group attachment and career adaptability was tested using Model 4 of the SPSS macro program PROCESS. As depicted in Figure 2, the overall impact of group attachment on career adaptability was found to be statistically significant (*β* = −0.27, SE = 0.05, 95% CI [−0.36, −0.18]). Additionally, the direct impact of group attachment on career adaptability was also significant (β = −0.15, SE = 0.05, 95% CI [−0.24, −0.06]), along with the significant indirect effect of perceived social support (*β* = −0.12, SE = 0.02, 95% CI [−0.17, −0.08]). This suggests that the connection between group attachment and career adaptability is partially mediated by perceived social support, accounting for 44.44% of the total effect.

### 3.4 Testing the moderated mediation model

**Figure 3:**
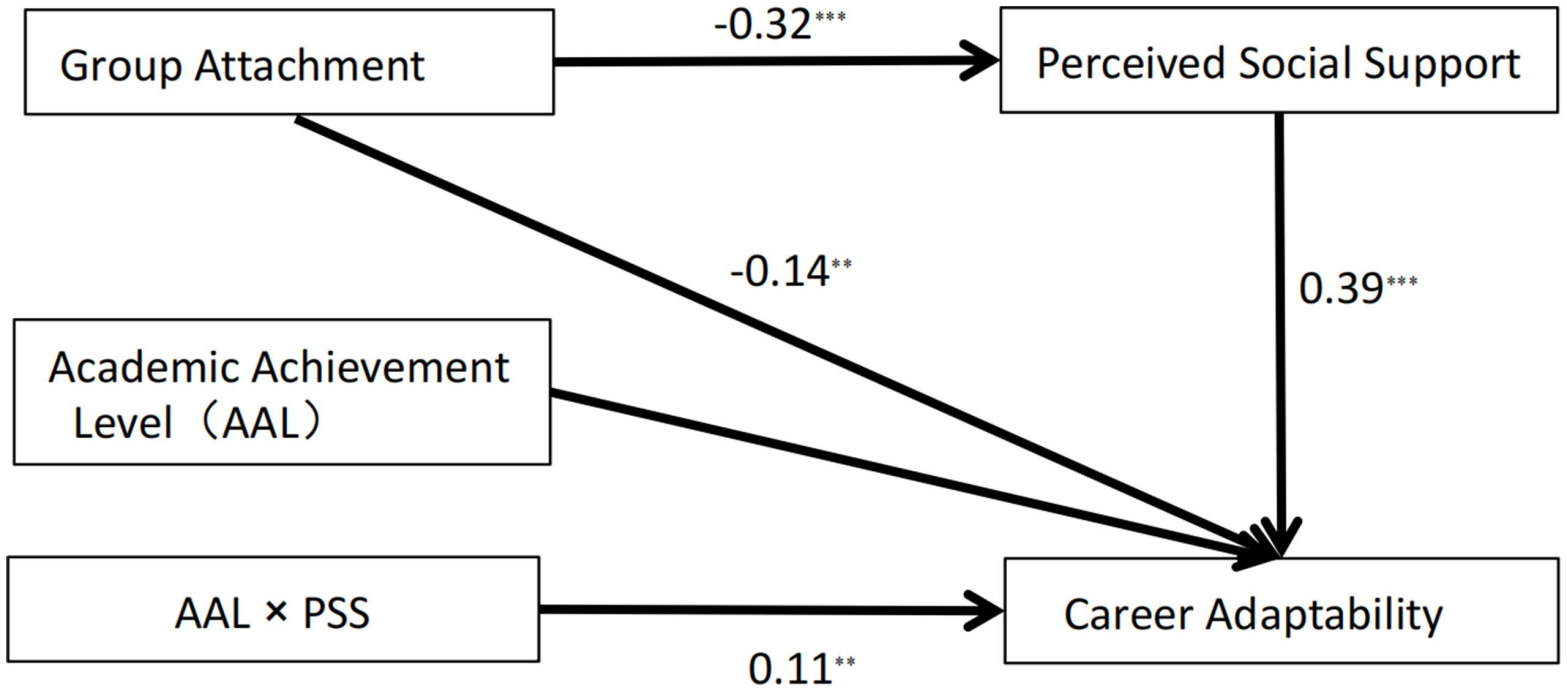
The moderating effect of Academic Achievement Level on the second stage of the indirect association. *p < 0.05, **p < 0.01, ***p < 0.001.

Moderated mediated effects were examined by employing Model 14 in the SPSS macro program PROCESS (refer to Figure 3). Regression analysis revealed that group attachment had a negative predictive impact on perceived social support (*β* = −0.32, *p* < 0.001). When both group attachment and perceived social support were included in predicting career adaptability, it was found that group attachment had a significant predictive effect (*β* = −0.14, *p* = 0.002), along with perceived social support (*β* = 0.39, *p* < 0.001). This indicates that perceived social support plays a partial mediating role in the relationship between group attachment and career adaptability.

When academic achievement level was taken into account, the interaction term between perceived social support and academic achievement level emerged as a significant positive predictor of career adaptability (*β* = 0.11, *p* = 0.008). This indicates that academic achievement level played a positive moderating role in the relationship between perceived social support and career adaptability. The mediating effect of group attachment on career adaptability through perceived social support was found to be significant, regardless of whether academic achievement was low (*β* = −0.09, SE = 0.02, 95% CI [−0.14,–0.05]) or high (*β* = −0.16, SE = 0.03, 95% CI [−0.22,–0.10]).

**Figure 4:**
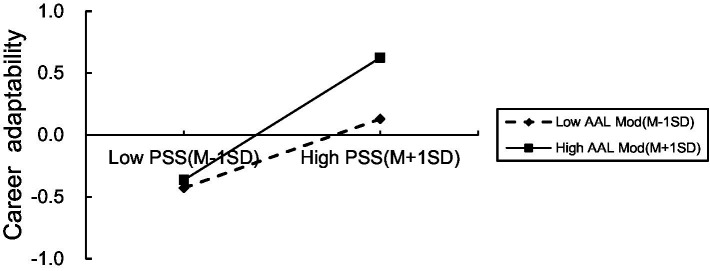
Interactive effect of perceived social support and academic achievement level on career adaptability.

In another simple slope test (refer to Figure 4), the impact of perceived social support on career adaptability was found to be significant when academic achievement was low moderated (Z = -1) (*B*_simple_ = 0.28, *t* = 4.81, *p* < 0.001, 95% CI of [0.17,0.39]). Conversely, the influence of perceived social support on career adaptability was even stronger when academic achievement level was high moderated (Z = 1) (*B*_simple_ = 0.49, *t* = 7.87, *p* < 0.001, 95% CI of [0.37, 0.62]). By showing that the level of academic achievement played a moderating role in the latter part of the mediating effect of perceived social support between group attachment and career adaptability, the inclusion of the interaction term enhanced the model’s ability to explain career adaptability by 1.2% (∆R2 = 0.012, *F* = 7, *p* = 0.008). The mediation effects differed significantly between the high and low academic achievement conditions, with a value of *β* = −0.07, SE = 0.03, and a 95% CI of [−0.12, −0.02]. There were no zeros in the middle, indicating a notable contrast in mediation effects.

## 4 Discussion

This study investigates the connection between group attachment and college students’ career adaptability, drawing on group attachment theory and career construct theory. Additionally, it explores how perceived social support partially mediates this relationship and how academic achievement level moderates the impact of perceived social support on career adaptability among college students. Exploring the underlying mechanisms, this study aimed to understand how group attachment influences the career adaptability of college students. The findings are crucial for higher education institutions to implement proactive measures in fostering college students’ career adaptability and improving their employability skills.

### 4.1 Group attachment has negative prediction on career adaptability

The research revealed that group attachment was inversely related to college students’ career adaptability, supporting Hypothesis 1. Prior research has shown a connection between college students’ group attachment and adaptability. For instance, a study involving nursing majors found that group attachment was inversely related to professional adaptability, with group attachment being a significant negative predictor of professional adaptability levels (Liu et al., 2018). Moreover, another study revealed that group attachment among college students negatively affected their school adjustment (Marmarosh and Markin, 2007), supporting the results of the present study. College students who have a secure group attachment are more likely to develop positive intra-group representational resources, receive emotional support, and psychological comfort from the group, which ultimately helps in promoting career adaptability. College students who have an insecure class group attachment tend to have negative intra-group representational resources, which can result in a decreased likelihood of receiving positive emotional and instrumental support from the group. College students who have high levels of group attachment anxiety tend to hesitate when faced with pressure due to fear of failure, depleting their psychological resources. On the other hand, college students with high levels of group attachment avoidance tend to have negative evaluations of the group and its members under pressure, avoiding seeking help from them, which weakens their social resources. Both of these behaviors can have a negative impact on the development of career adaptability. Additionally, research has verified that the internal working model of the group among college students directs them to assimilate the group’s culture, impacting their performance and adaptation within the group (Cortina and Marrone, 2004). This discovery underscores the significance of group attachment in predicting the adaptability of college students. It provides insights into the correlation between group attachment and career adaptability among college students, advances the study of group attachment theory in the realm of career adaptability, and enhances the understanding of factors that impact career adaptability.

### 4.2 The mediating role of perceived social support

The study revealed a significant negative correlation between group attachment and perceived social support. Attachment and perceived social support have been identified as key factors for career adaptability in previous research conducted by Ramos and Lopez (2018), Zhang (2019), and Rençber and Paşaoğlu Baş (2023), supporting the findings of this study. Wang et al. (2022) conducted an interview survey study on 12 college graduates using the rootedness theory. They discovered that situational factors, like lack of social support, had a significant impact on subjective career unsuccessfulness and decreased career adaptability levels. These findings align with the results of the current study. The current study revealed that group attachment had a significant negative impact on perceived social support, aligning with previous findings (Smith et al., 1999). This supports the idea that perceived social support serves as a partial mediator in the relationship between group attachment and career adaptability, thus confirming Hypothesis 2. Ye et al. (2023a) proposed that adaptive readiness for career adaptability comprises cognitive, affective, and behavioral elements. It has been suggested that a serial relationship exists between adaptive readiness factors. Du et al. (2022) discovered that core self-evaluation and protean career attitudes both have a significant impact on career adaptability. There is a sequential relationship where core self-evaluation leads to protean career attitudes, which then influences career adaptability. The study also validated the sequential relationship between group attachment and perceived social support in influencing career adaptability as “group attachment-perceived social support-career adaptability.” Additionally, it confirmed the sequential relationship between adaptive readiness as “adaptive readiness 1-adaptive readiness 2.”

### 4.3 The moderating role of academic achievement level

The study’s findings also indicated that the level of academic achievement played a role in influencing the connection between perceived social support and career adaptability, thus supporting Hypothesis 3. This is consistent with the findings of previous studies conducted by Ye et al. (2014), Datu and Buenconsejo (2021), and Rimša et al. (2023). The current research discovered that the correlation between perceived social support and academic achievement level had a significant and positive impact on career adaptability, particularly when the academic achievement level was low. Moreover, this correlation was further strengthened when the academic achievement level was high. The results support the facilitation hypothesis of the “protective factor-protective factor model” within the framework of the enhancement model of interaction (Wang et al., 2016). Under the same level of perceived social support, college students with high levels of academic achievement have stronger self-concepts, competence levels, and learning abilities, and are more likely to obtain instrumental, emotional, informational, and peer support from their environments, which enhances college students’ adaptive ability to cope with stressful situations. This discovery reinforces the connection between academic achievement level and college students’ career adaptability. When examining the factors that contribute to the development of career adaptability, it is important to consider how individual growth is shaped by the interplay between external factors, such as academic achievement, and internal factors, such as perceived social support. To enhance the positive impact of perceived social support on career adaptability, it is essential to create an environment where individuals feel a greater sense of mastery over their abilities.

### 4.4 Limitations and future directions

Moreover, this research has the following limitations. Although the cross-sectional study was unable to confirm the mechanism through which group attachment influences career adaptability, a potential solution could be to conduct a follow-up study to determine the order of influence between group attachment and social support on career adaptability. Additionally, having an insecure adult attachment can have a strong negative impact on one’s career adaptability. It is suggested that adult attachment and group attachment may interact with each other in influencing college students’ career adaptability, following the “risk factor-risk factor” model. Future research should delve into the potential interaction between adult attachment and group attachment on career adaptability. In the future, researchers can investigate how adult attachment and group attachment interact to influence career adaptability, while also examining the impact of group attachment on career adaptability among college students after accounting for insecure adult attachment. In conclusion, this research relied on participants’ self-reports, and moving forward, information will be gathered using teacher and peer evaluations to confirm the research inquiries.

### 4.5 Theoretical contributions and practical implications

The study findings uncover how group attachment impacts college students’ career adaptability. This finding enhances the understanding of the factors that influence college students’ career adaptability, expands the application of attachment theory in the context of career adaptability, and responds to the call from scholars to conduct research from a psychological perspective to enhance college students’ school adaptation and promote high-quality all-round development (Ye and Chen et al., 2024). Higher education institutions can enhance the cultivation of positive psychological qualities and career adaptability in college students by improving class group construction at a practical level. Schools and classes play a crucial role in shaping the physical and mental well-being of college students, as well as influencing their employability (Li and Xu, 2022). Colleges and universities have the option to implement various strategies and utilize a multifaceted approach when it comes to providing career adaptability education. Standardized assessment tools can help identify the adaptive readiness of various college student groups, such as group attachment. By using these tools, targeted activities like class group counseling and thematic class meetings on career development can be implemented to improve the emotional connection college students have with their class groups. Conversely, gaining a better understanding of the distal and proximal factors that influence career adaptability can give career counselors in colleges and universities guidance on how to improve career adaptability in a practical way. This research discovered that perceived social support acts as a mediator between group attachment and career adaptability, offering valuable insights for colleges and universities to implement various interventions to improve career adaptability. Higher education institutions can promote the development of college students’ career adaptability and enhance their lifelong learning abilities by building a positive social support network and providing precise academic assistance. This helps achieve the positive talent cultivation goals of higher education. Colleges and universities can conduct surveys to assess career development needs in various ways, offering personalized career counseling and employment guidance to students. This not only helps students enhance their career adaptability, but also improves the professional diagnosis and assistance skills of staff involved in employment services, ultimately increasing the effectiveness of employment support.

The findings of this research underscore the significance of academic success in shaping the career adaptability of college students. The influence of perceived social support on career adaptability is contingent upon the level of academic achievement. College students’ academic performance can shape their aspirations for advanced education and career choices, as well as impact their intentions for future employment (Cai and Da, 2023). Vuolo et al. (2014) highlighted how academic achievement can enhance the transition from school to work. High-achieving college students tend to receive more support from their social networks, such as family, friends, and teachers, which helps them accumulate psychological and social resources for career development. This support enables them to invest more time and energy in planning for their future careers, ultimately enhancing their career adaptability compared to their low-achieving peers. Colleges and universities should prioritize providing college counseling services not only to students with high academic achievement, but also to those from low academic achievement groups. It is important to offer group or individual counseling activities to help these students identify their strengths and resources. Additionally, colleges should enhance the promotion of their career guidance services and support systems to increase students’ awareness of available social support. By providing students with the necessary psychological and social resources for career development, colleges can help improve their career adaptability.

## 5 Conclusion

This research expanded on the existing literature regarding the influences on career adaptability among college students at the campus level. It explored the role of group attachment in enhancing career adaptability from a psychological perspective and confirmed the predictive power of the career construct theory “Adaptive Readiness 1-Adaptive Readiness 2” on career adaptability. Insecure group attachment was found to significantly and negatively predict career adaptability. This relationship was mediated by perceived social support, with academic achievement level playing a moderating role in the second half of the mediation pathway. Perceived social support at high levels can help alleviate the negative consequences of insecure group attachment on career adaptability. Moreover, the protective influence of perceived social support increases in strength as academic achievement levels rise. It is suggested that enhancing class group formation, establishing a strong social support network, and implementing a varied academic achievement assessment system are key methods to enhance college students’ career adaptability.

## Data Availability

The original contributions presented in the study are included in the article/supplementary material, further inquiries can be directed to the corresponding author/s.
